# Nomogram Based on Liver Function Test Indicators for Survival Prediction in Nasopharyngeal Carcinoma Patients Receiving PD-1 Inhibitor Therapy

**DOI:** 10.3390/curroncol30080521

**Published:** 2023-07-26

**Authors:** Lixia Liang, Yan Li, Yansui Hong, Tianxing Ji, Hao Chen, Zhifang Lin

**Affiliations:** 1 Department of Clinical Laboratory, The First People’s Hospital of Zhaoqing, Zhaoqing 526060, China; lianglixia3@zqmc.edu.cn (L.L.); iamyanlee@163.com (Y.L.); 15767322327@163.com (Y.H.); 2 Clinical Laboratory Medicine Department, The Second Affiliated Hospital of Guangzhou Medical University, Guangzhou 510060, China; jitianxing7021@163.com; 3 Department of Clinical Laboratory, State Key Laboratory of Oncology in South China, Collaborative Innovation Center for Cancer Medicine, Guangdong Key Laboratory of Nasopharyngeal Carcinoma Diagnosis and Therapy, Sun Yat-Sen University Cancer Center, Guangzhou 510060, China

**Keywords:** nasopharyngeal carcinoma, PD-1 inhibitors, liver function tests, metastasis stage, pretreatment nomogram

## Abstract

Purpose: The aim of this study was to investigate the prognostic significance of PD-1 inhibitor therapy in nasopharyngeal carcinoma (NPC) and to develop a nomogram to estimate individual risks. Methods: We retrospectively analyzed 162 NPC patients who were administered the PD-1 inhibitor combined with radiotherapy and chemotherapy at the Sun Yat-Sen University Cancer Center. In total, 108 NPC patients were included in the training cohort and 54 NPC patients were included in the validation cohort. Univariate and multivariate Cox survival analyses were performed to determine the prognostic factors for 1-year and 2-year progression-free survival (PFS). In addition, a nomogram model was constructed to predict the survival probability of PFS. A consistency index (C-index), a decision curve, a clinical impact curve, and a standard curve were used to measure predictive accuracy, the clinical net benefit, and the consistency of prognostic factors. Results: Univariate and multivariate analyses indicated that the metastasis stage, the levels of ALT, the AST/ALT ratio, and the LDH were independent risk factors associated with the prognosis of PD-1 inhibitor therapy. A nomogram based on these four indicators was constructed and the Kaplan–Meier survival analysis showed that patients with a higher total score have a shorter PFS. The C-index of this model was 0.732 in the training cohort and 0.847 in the validation cohort, which are higher than those for the TNM stages (training cohort: 0.617; validation cohort: 0.727; *p* <0.05). Decision Curve Analysis (DCA), Net Reclassification Improvement (NRI), and Integrated Discrimination Improvement (IDI) showed that our model has better prediction accuracy than TNM staging. Conclusions: Predicting PFS in NPC patients based on liver function-related indicators before PD-1 treatment may help clinicians predict the efficacy of PD-1 treatment in these patients.

## 1. Introduction

Nasopharyngeal carcinoma (NPC) is a type of cancer wherein the tumor develops in the epithelium of the nasopharynx. NPC is widespread throughout in Southeast and East Asia, with Southeast China having the highest incidence of 20–50 cases per 100,000 persons [1,2]. Radiation treatment and concurrent adjuvant chemotherapy have led to an improvement in the clinical outcomes of NPC. However, tumor recurrence and distant metastasis remain challenges, occurring in 20% to 30% of patients [3,4]. Patients with metastatic/recurrent NPC have poor prognosis and platinum-based palliative chemotherapy is the only treatment option for them [5]. T-cell checkpoint inhibitors, such as the anti-cytotoxic T-lymphocyte-associated protein 4 (CTLA4), monoclonal antibody, such as ipilimumab, and the anti-programmed cell death protein 1 (PD-1) monoclonal antibodies, including pembrolizumab and nivolumab, have improved patient prognosis in a number of cancer types [4]. This anti-PD-1 immunotherapy has only been used in clinical treatment in recent years, and its use in the field of NPC treatment is relatively nascent. The immune checkpoint inhibitor anti-PD-1 immunotherapy has recently become a popular therapy option for malignancies [6]. The paradigm for cancer therapy has been completely altered by blocking the PD-1/PD-L1 inhibitory immune checkpoint. Thus, this particular checkpoint pathway has been the focus of extensive research. In 2020, the Chinese Society of Clinical Oncology (CSCO) guidelines officially recommended first-line and second-line therapy of recurrent/metastatic NPCs with the PD-1 inhibitor. In addition, first-line clinical trials for the treatment of locally advanced nasopharyngeal cancer (before, during, or after radiotherapy) are also being widely performed. Preliminary results indicate that the PD⁃1 inhibitor has good application prospects [7]. Early studies have shown clinical efficacy and good safety of targeted therapy and immunotherapy in NPC, but many studies are only in the preclinical or early stage of research, and more phase III clinical trials are needed. Targeted therapy and immunotherapy have the potential to become adjuvant therapies, whereas monotherapy has limitations; a combination of these therapies with conventional therapy is likely the future trend.

The liver facilitates immunological tolerance in autoimmune illnesses, viral infections and organ transplantation, and it is a frequent target for cancer metastases [8,9]. Uncertainty persists regarding the significance of these liver immune tolerance systems in relation to cancer. Liver metastasis is associated with lower response rates, progression-free survival, and overall survival according to the growing body of research [10,11,12,13,14,15,16]. Given that all cancers commonly metastasize to the liver, this issue results in significant unresolved difficulty in the study of immuno-oncology [10,13,16,17]. The nomogram has been acknowledged as a trustworthy predictor of the individual numerical outcomes since it combines pathological, clinical, and therapy-related characteristics into a statistical model, indicating the likelihood of clinical outcomes in cancer [18]. According to the most recent study, nomograms can assist in achieving a more accurate prognosis than the TNM classifications currently used in clinical practice for a number of cancers types [19,20,21]. It has been reported that parotid lymph node metastasis, early heterochronous metastasis, and nomograms based on clinical characteristics and the prognostic effects of blood markers in NPC patients bring better prognostic accuracy in clinical practice [22,23,24]. Tumor treatments are becoming increasingly accurate and individualized. Biomarkers will become road signs on the path of developing targeted therapy and immunotherapy. Predictive models need to be established so that a comprehensive evaluation of predictive factors can be more accurately used to select beneficiary groups. However, to date, a nomogram has been developed that can predict an NPC patient’s prognosis prior to PD-1 inhibitor therapy. Based on these presumptions, we created a nomogram of liver function test (LFT) indicators used with PD-1 inhibitor therapy to predict a patient’s prognosis in NPC. The outcomes of this nomogram can be used to direct customized treatment.

## 2. Materials and Methods

### 2.1. Study Population and Study Design

This research was observational in nature. In total, 162 NPC patients who received PD-1 inhibitors for the first time at the Sun Yat-Sen University Cancer Center from 23 March 2018 to 6 July 2020, were randomly divided into two groups (ratio 2:1). There were 108 NPC patients in the training cohort which was used to construct the predictive model, and there were 54 NPC patients in the validation cohort which helped validate the model. Diagnosis in cases of NPC was assigned using spiral CT combined with histopathology.

Patients who met the following criteria were included in the analysis: (1) a histopathological examination confirmed a diagnosis of NPC, (2) diagnosis щa NPC before PD-1 inhibitor treatment of PD-1, diagnosis of NPC, and treatment with radiotherapy or chemotherapy or both, (3) complete follow-up information, (4) consent for undergoing PD-1 inhibitor therapy, and (5) absence of liver resection or transplantation. PFS was outlined as the period of time from randomization to a patient’s tumor progression or death. The final follow-up was conducted on May 9, 2021. The treatment for NPC in this trial followed the Chinese Society of Clinical Oncology (CSCO) guidelines. The Sun Yat-Sen University Cancer Center’s ethical committee approved this research (http://www.researchdata.org.cn, accessed on 31 May 2022, RDDA2022826869). Informed consent was obtained from all participants. The guiding principles of the Declaration of Helsinki were applied to every aspect of the work. 

The AJCC TNM staging manual was used to determine the clinical staging of NPC, 8th edition. Physical examination, nasopharyngeal endoscopy, nasopharyngeal and neck (plain scan and enhanced) MRI and CT, neck ultrasound, chest and abdomen CT or abdominal ultrasound, whole body bone scan, and other imaging examinations were conducted depending on the medical history and clinical manifestations of the patient to assess the degree of local lesion invasion, the status of lymph node metastasis, and the presence of distant metastasis, and to accurately obtain TNM staging information. If the patient was unable to undergo an MRI examination for some reason, a CT examination was performed to identify the extent of the nasopharyngeal primary lesions. PET-CT was used to rule out latent distant metastases in individuals with high risk factors for the disease (stage T4 and/or N2-3).

Serum test data included those for the liver function index levels (alanine aminotransferase (ALT), aspartate aminotransferase (AST), ratio of AST/ALT, total protein (TP), albumin (ALB), total bile acid (TBA), cholinesterase (CHE) levels, alkaline phosphatase (ALP), gamma glutamyltransferase (GGT), and total bilirubin (TBIL)), renal function index levels (UREA, creatinine (CREA), uric acid (UA), and glucose (Glu)), cardiac enzyme index levels (lactate dehydrogenase (LDH), and creatine kinase (CK)), and lipid index levels (triglycerides (TG), cholesterol (CHO), high-density lipoprotein-C (HDL), low-density lipoprotein-C (LDL), apolipoprotein A1 (ApoA1), and apolipoprotein B (ApoB)). These data were obtained for this study both before and three weeks after the commencement of treatment with PD1 inhibitors.

An initial assessment of efficacy was made at 8–12 weeks and is continuously thereafter. Baseline covariate data were collected, including information on age, sex, body mass index (BMI), smoking and drinking history, clinical stage, histological type, recurrence, TNM typing data, and PD-1 blockade types. Radiological examinations were performed according to RECIST (Solid Tumor Response Assessment Criteria) v1.1 to evaluate the effects of 8–12 weeks of immunotherapy in the training and validation cohorts, and the following parameters were evaluated: complete response (CR), partial response (PR), stable disease (SD), and progression disease (PD). The time interval between the start date of treatment with PD-1 inhibitors and the date of disease progression or death (PFS) was calculated for each patient.

### 2.2. Statistical Analysis

The SPSS25.0 (Chicago, IL, USA) and R (4.1.3) software were utilized for statistical analysis. To evaluate clinical and pathological features, univariate analysis was performed. The factors with a significance of *p* < 0.05 in univariate analysis were further evaluated via multivariate Cox regression analysis. To assess the impact of the prognostic variable PFS, Hazard Rratio (HR) and 95% confidence interval (CI) were calculated using univariate and multivariate Cox proportional risk regression models. To calculate cutoff values for numerical variables in the training cohort, the maximally selected rank statistics via the “surv_cutpoint” function of the “survminer” R package were used, and the cutoff values were verified by MedCalc software. Kaplan–Meier survival analysis was performed to plot the survival curves, and the “*survminer*” and “*survival*” R packages were used to compare them with a logarithmic rank test. In the multivariate model, a dynamic prediction nomogram was established using all variables with *p* < 0.05. By comparing predicted and observed survival, the prognostic factors of PFS were calibrated for 1 to 2 years.

## 3. Results

### 3.1. Patient Characteristics

In total, 162 NPC patients met the inclusion criteria. The cut-off value for age was 42 years, and 102 of 162 patients (63.0%) were aged more than 41 years. In total, 115 were males (71.0%) and 47 were females (29.0%). Further, 41 (25.3%) and 19 (11.7%) patients were current or former smokers and drinkers, respectively. Moreover, 153 cases (94.4%) were undifferentiated, and 9 (5.6%) were poorly differentiated. The tumor stages of T0–T2 were assigned to 38 cases (23.5%) and those of T3–T4 were assigned to 124 cases (76.5%). Node stages of N0–N1 were assigned to 70 cases (43.2%) and those of N3–N4 were assigned to 92 cases (56.8%). Further, 77 cases (47.5%) were of metastasis stage M0 and 85 (52.5%) were of metastasis stage M1. With regard to distant metastasis to one or multiple sites, 63 cases showed distant lymph node metastasis (38.9%); 26 showed liver metastasis (16.1%); 36 showed lung metastasis (22.2%); and 45 showed bone metastasis (27.8%). Recurrence before PD-1 inhibitor treatment was observed in 74 cases (45.7%). The PD-1 inhibitor camrelizumab was used in 18 cases (11.1%), toripalimab was used in 119 cases (73.5%), pembrolizumab was used in three cases (1.9%), and sintilimab was used in 22 cases (13.5%). PD-1 inhibitor combination therapy drugs included gemcitabine, paclitaxel, cisplatin, carboplatin, apatinib, nimotuzumab, and 5-fluorouracil. At the last follow-up, the median PFS was 85 days. Further, 45.7% of patients developed a progressive disease, and 54.3% of patients maintained disease control among the total cohort. The demographic and clinical characteristics of all patients, training cohort, and validation cohort are summarized in Table 1. 

### 3.2. Univariate and Multivariate Cox Proportional Hazard Regression Analysis for PFS

The best truncation values for all variables included age (>41 years), BMI (>22.3), ALT (≤14.3 U/L), AST (>25.3 U/L), AST/ALT ratio (>1.3), CHE (≤7906 U/L), ALP (≤99.2 U/L), GGT (≤15.9 U/L), TBA (>2.3 μmol/L), TBIL (≤10.2 μmol/L), DBIL (≤1.8 μmol/L), TP (≤75.5 g/L), ALB (≤38.8 g/L), Urea (≤4.4 mmol/L), CREA (≤55.3 μmol/L), UA (≤379.8 μmol/L), Glu (≤4.84), LDH (>203.0 U/L), CK (≤36.0 U/L), TG (>1.07 mmol/L), CHO (>4.91 mmol/L), HDL (>0.98 mmol/L), LDL (>2.95 mmol/L), ApoA1 (>1.11 g/L), and ApoB (>1.21 g/L). All indices were analyzed by univariate analysis; when an index showed *p* <0.05, multivariate Cox regression analysis was performed. After multivariate COX regression analysis, the indices with *p* < 0.05 were identified to be potential risk factors. We found that the potential prognostic factors included the metastasis stage, ALT, AST/ALT, TBA, and LDH in the training cohort.

Univariate analysis showed that the metastasis stage (*p* = 0.001), ALT (*p* = 0.006), AST/ALT ratio (*p* = 0.001), TBA (*p* = 0.041), and LDH (*p* = 0.001) were significantly associated with PFS. Other clinical and biochemical indices included tumor and node stage (*p* > 0.05). In addition to the clinical case characteristics, 48 patients were tested for HAV, HCV, and HEV, all of which were negative. HBsAg detection was performed in 93 of the 162 patients, with a positive rate of 9/93 (9.7%) (*p* > 0.05). Further, EBV-DNA detection was performed in 12 patients, with a positive rate of 4/12 (33.3%). 

**Figure 1:**
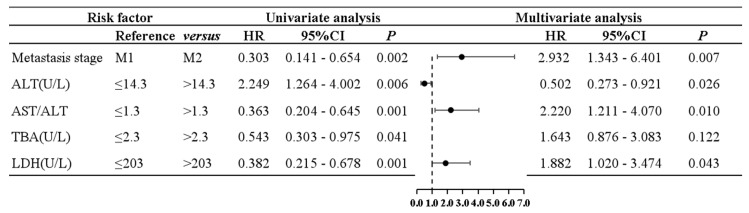
A grouped forest plot of univariate and multivariate Cox proportional hazards regression analysis for PFS.

Next, in multivariate Cox proportional risk regression analysis for PFS, the abovementioned prognostic factors were included. Results of multivariate analysis showed that the following were independent risk factors: the metastasis stage (*p* = 0.007, HR = 2.932, 95%CI: 1.343–6.401), ALT (*p* = 0.026, HR = 0.502, 95%CI: 0.273–0.921), AST/ALT ratio (*p* = 0.010, HR = 2.220, 95%CI: 1.211–4.07), and LDH (*p* = 0.043, HR = 1.882, 95%CI: 1.020–3.474). According to the univariate and multivariate analysis and the Cox proportional risk regression, a grouped forest plot was constructed, showing risk ratios and 95% confidence intervals for PFS. Univariate and multivariate analyses and grouped forest plots showed risk ratios and 95% confidence intervals for PFS according to the Cox proportional risk regression (Figure 1).

### 3.3. Survival Analysis

**Figure 2:**
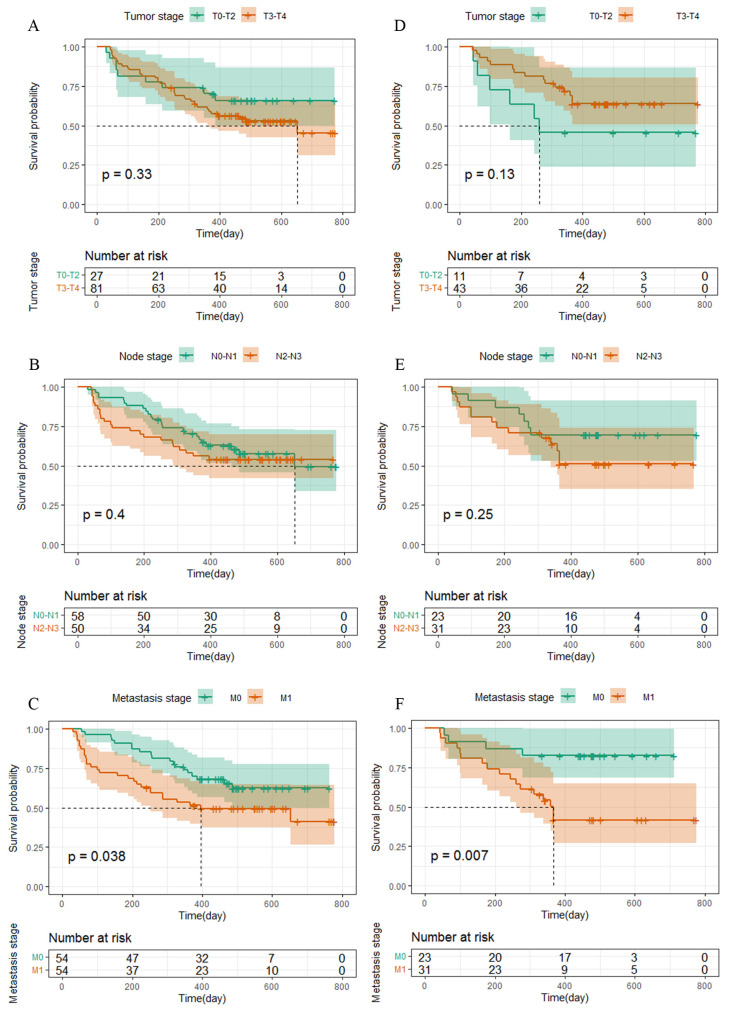
Kaplan–Meier curves for PFS in NPC patients. (**A**–**C**) The TNM stage in NPC patients in the training cohort were plotted as a distribution. (**D**–**F**) The TNM stage in NPC patients in the validation cohort were plotted as a distribution.

**Figure 3:**
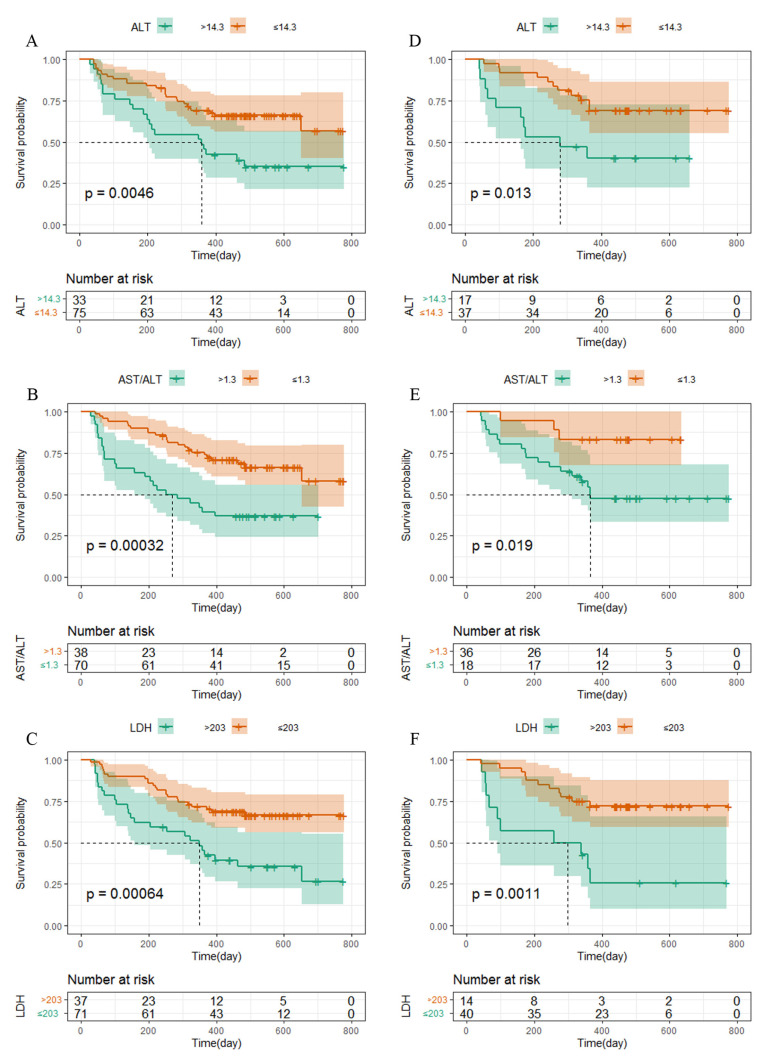
Kaplan–Meier curves for PFS in NPC patients. (**A**–**C**) The metastasis stage, ALT, AST/ALT, and LDH in NPC patients in the training cohort were plotted as a distribution. (**D**–**F**) The metastasis stage, ALT, AST/ALT, and LDH in NPC patients in the validation cohort were plotted as a distribution.

The survival curve was drawn using Kaplan–Meier survival analysis, and significant differences were shown for the level of the TNM stage (Figure 2), ALT, AST/ALT ratio, and LDH, among other independent influencing factors. The logarithmic rank test confirmed that the training cohort’s and validation cohort’s results were similar (Figure 3).

### 3.4. Constructing Nomogram

**Figure 4:**
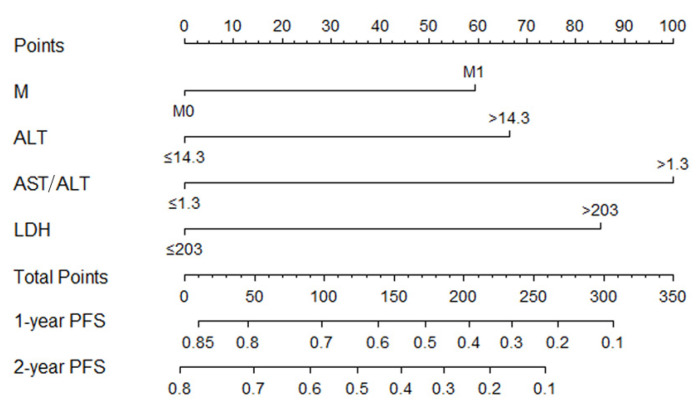
Nomogram model based on metastasis stage (M), ALT, AST/ALT, LDH in the prediction of 1-, 2-year PFS in NPC patients. The nomogram was used by summing the points identified on the point scale for each variable. The total points projected on the bottom scales indicate the probability of 1- and 2-year survival.

Based on multivariate analysis, a nomogram was constructed for PFS prediction and involved all independent prognostic factors, including the metastasis stage, ALT, AST/ALT ratio, and LDH (Figure 4). The multiple risk points associated with each prognostic factor can be obtained by directly plotting a vertical line-up from the value corresponding to the prognostic factor all the way to the point axis. A vertical line may be drawn on an axis labeled “1-year and 2-year progression-free survival probabilities” to evaluate the 1-year and 2-year PFS probabilities for a particular patient from the “total integral” of the sum of risk points. The nomogram shows that AST/ALT ratio has the greatest effect on PFS, followed by LDH, ALT, and the metastasis stage. A higher total score in the nomogram indicates a shorter PFS.

**Figure 5:**
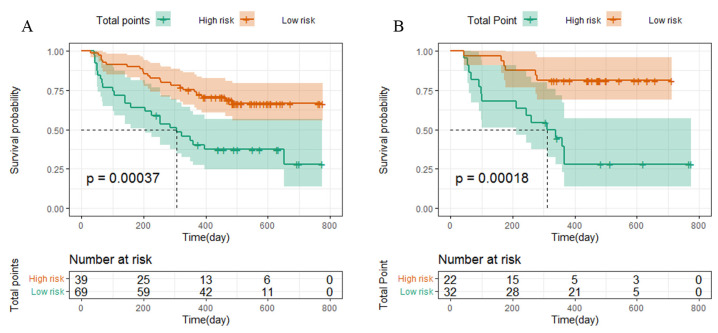
Kaplan–Meier curves for PFS based on predictions of the nomogram. PFS (**A**) in the training cohort and PFS (**B**) in the validation cohort. Low risk: total points ≤ 145; High risk: total points > 145.

Based on the cut-off values of the total points determined by the R (4.1.3) software, patients were subdivided into a low-risk group (total points ≤ 145) and a high-risk group (total points > 145) for PFS in the training and validation cohorts. Kaplan–Meier survival analysis was performed to predict each group’s survival duration. The median PFS in the training cohort was 308.8 days for high-risk patients and 453 days for low-risk patients (*p =* 0.00037; Figure 5A). Patients in the high-risk group in the validation cohort had a shorter PFS (308.3 days) than those in the low-risk group (431.9 days, *p* = 0.00018, Figure 5B). 

### 3.5. Nomogram Accuracy and Calibration

**Figure 6:**
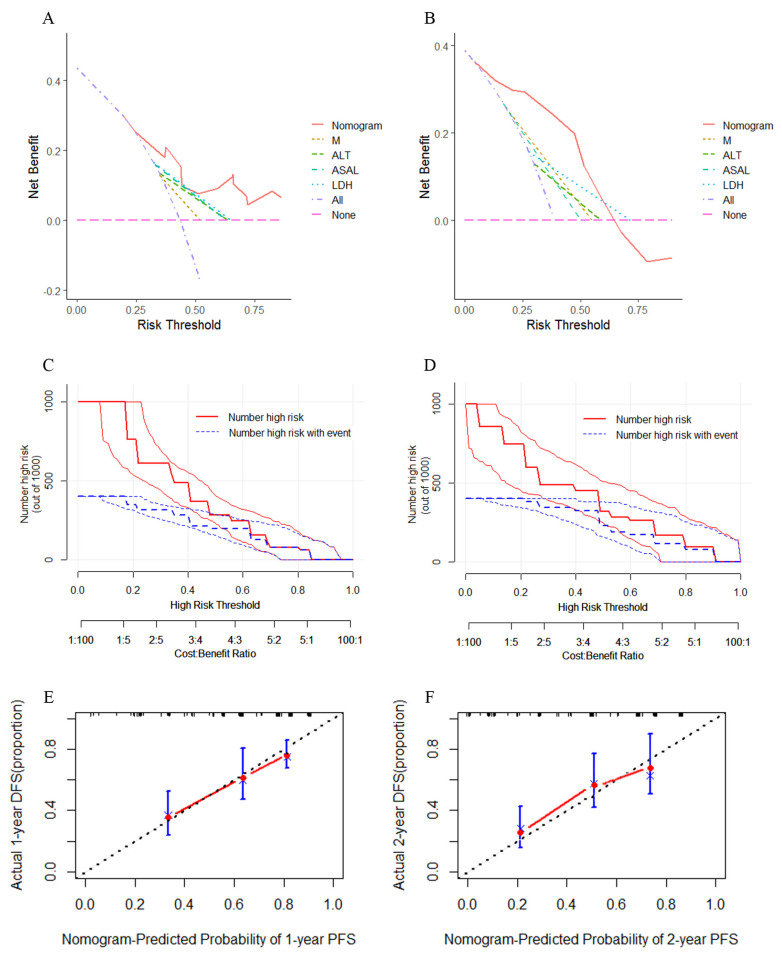
The Decision Curve for the training cohort (**A**) and the validation cohort (**B**) of PFS in NPC patients. The Clinical impact curve for the training cohort (**C**) and the validation cohort (**D**). The Calibration curves of the Nomogram-Predicted probability of 1-year PFS (**E**) and Nomogram-Predicted probability of 2-year PFS (**F**).

The C-index of this model within the training cohort was 0.732 (95%CI: 0.540–0.924), which was higher than the C-index of individual prognostic factors and TNM stages (C-index = 0.617, 95%CI: 0.411–0.823). The C-index was 0.847 (95%CI: 0.545–1.049) for the validation cohort, both of which were higher than the accuracy of individual prognostic factors and TNM staging (C-index = 0.727, 95%CI: 0.462–0.992). There were statistically significant differences between the nomogram for the training cohort and that for the validation cohort with a single factor and TNM stage when compared pairwise (*p* < 0.05; Table 2). To assess the net benefit of the nomogram, we combined the training and validation cohorts, and then performed Decision Curve Analysis (DCA), Net Reclassification Improvement (NRI), and Integrated Discrimination Improvement (IDI) to evaluate prediction accuracy. The DCA curves of both the training (Figure 6A) and validation cohorts (Figure 6B) demonstrated that the index (solid red line) had higher net benefit in predicting both 1-year and 2-year PFS compared with other individual prognostic factors and TNM staging. We also used the nomogram model for predicting risk stratification of 1000 people, showing “loss”. The clinical impact curves of the training cohort (Figure 6C) and the validation cohort (Figure 6D) were obtained by using eight ratio scales. The blue curve shows how many people are actually positive at each probability threshold, whereas the red curve shows how many people the model classified as positive (high risk) for each probability. Moreover, this demonstrates that the nomogram prediction model for the cohort has a larger net benefit clinically after being trained and validated. Both the 1-year (Figure 6E) and 2-year (Figure 6F) PFS calibration curves demonstrate satisfactory calibration, indicating agreement between nomogram-based prediction and actual observation. The NRI of both the training cohort and the validation cohort indicated a positive improvement (NRI > 0), signifying that the nomogram model is more accurate in predicting events than individual prognostic factors and TNM stages. Furthermore, IDI demonstrated that compared with both individual prognostic factors and the TNM stage (IDI > 0), the nomogram showed improved accuracy in predicting PFS. Overall, the newly established nomogram has better net benefit and accuracy (Table 3).

## 4. Discussion

Approximately 70% of unsuccessful treatments are followed by distant metastases in NPC [25]. For metastasized NPC, the risk cannot only be determined by TNM classification. Therefore, it is urgent to establish a predictive model with a more accurate prediction ability. In this study, we found that metastasis stage, ALT, AST/ALT ratio, and LDH were independent predictors of PFS in NPC patients undergoing PD-1 inhibitor therapy. Here, we established a nomogram based on LFTs and the metastasis stage to assess their relationship with PFS in NPC patients undergoing PD-1 inhibitor therapy, and compared the predictive power of our model with traditional TNM staging systems using C-index, DCA, NRI, and IDI. The nomogram demonstrates satisfactory prediction and calibration capabilities. As observed, this is the first nomogram showing survival results among NPC patients treated with PD-1 inhibitor based on indicators related to LFTs. As shown by multivariate analysis, this nomogram can better predict the PFS based on LFTs indices and the metastasis stage in patients undergoing combination treatment with the PD-1 inhibitor. Our study showed that the nomogram showed better accuracy than the TNM staging system, which might facilitate individualized prediction for future consultations.

LFTs are a common clinical means for detecting liver disease, and they have been used to detect serum enzyme activity during liver injury in clinical practice. When the liver is inflamed or subjected to any other damage, liver cell metabolism and cell membrane permeability change, causing liver enzymes to be released into the blood. These enzymes can then be detected in the serum, such as indicators, to assess the liver damage degree and severity of liver disease [26]. In addition, LFTs are a first-line clinical indicator for detecting drug-induced liver injury.

ALT is the most sensitive index with regard to liver damage and is therefore most commonly used for reflecting liver function. When any condition causes liver injury, the liver cell membrane bursts, and transaminase can be released into the blood, causing transaminase levels to rise. However, apart from the liver cells, cardiac muscle and skeletal muscle cells also have transaminase. ALT can only detect the activity of liver cells, and cannot be used to diagnose liver cancer. When there is inflammation in the liver, ALT in the liver cells can be diffused out of the cell and transferred into the blood, resulting in an increase in ALT levels when the blood test is performed, thereby leading to a hepatitis diagnosis. However, in the early stages of liver cancer, there are no obvious abnormalities in liver function. Furthermore, liver cells only show certain inflammation and death because of the production of cancer cells. When liver cancer gradually develops and cancer cells develop outside the liver, liver dysfunction can occur, and the ALT flows outside and becomes absorbed by the blood. ALT is an enzyme that is released into the blood after the death of liver cells. Accordingly, when liver cancer is at an advanced stage, there are few normal cells in the liver, so ALT becomes normal again, which indicates poor prognosis. Hence, more attention needs to be paid at this time.

Many studies have confirmed that the levels of ALT, AST and their ratio LST (ALT/AST) are associated with many cancer types, including liver cancer, kidney cancer, colon cancer, non-small cell carcinoma, breast cancer, and pancreatic cancer [27,28,29,30,31,32]. Shen et al. showed that patients with high HBV-DNA loads and AST/ALT ratios >1 had less CD8+ T cell infiltration and higher levels of PD-L1 expression. High HBV-DNA loads and AST/ALT ratios may be a sign of acute hepatic inflammation, which may promote the development of hepato-carcinogenesis and tumor spread [33].

LDH has also been recognized as a useful pre-diagnostic and/or prognostic biomarker in various cancers. The most frequent means through which LDH controls cell invasion and migration was through lactate secretion. The prevalence of distant metastasis and the rate of early distant cancer metastasis have both been linked to lactic acid levels. The motility of tumor cells and the haphazard migration of many cell types can both be facilitated by exogenous lactic acid. It has been shown that lactate influences cell motility, promoting interactions between cancer cells and stromal cells to encourage angiogenesis and metastasis [34]. Neoadjuvant chemotherapy combined with radiation and high serum LDH activity level are independent risk factors that negatively affect overall survival (OS) in patients with locally advanced NPC [35,36]. LDH is regarded as a reliable indicator of survival in patients with invasive lymphocytic carcinoma and is one of the risk variables in the International Prognostic Index (IPI) [37]. We showed that the AST/ALT ratio and LDH dynamic alterations were associated with the effectiveness of the PD-1 inhibitor in NPC.

Immune-related adverse events (irAE) affecting the skin have increased treatment with PD-1 or the PD-L1 inhibitor; liver, gastrointestinal tract, and endocrine glands have also become affected. Hepatotoxicity after treatment with immune checkpoint inhibitors (ICI) is rare, but a potentially fatal AE is clinically significant. Although many cases are mild or asymptomatic and hardly much action is needed, certain cases that are jaundice-free at first may progress and become life-threatening without proper treatment [38,39]. Unfortunately, in the clinical literature, there is no consensus regarding the definition of immune-associated hepatitis. Distinguishing immune-associated hepatitis from other causes of liver injury is therefore difficult. Although immune checkpoint inhibitor-related hepatotoxicity (ICH) is mainly considered to be "hepatitis" of transaminase elevations, its definition usually includes immune-mediated cholangitis and biliary enzyme rise. The diagnosis is further complicated by ICI increasingly being used in combination with various chemotherapies and targeted treatments. Hepatotoxicity may result from these treatments. irAEs associated with treatment are manageable, and close monitoring for liver toxicity is recommended [40]^.^ In fact, because of co-dosing medication, it could be difficult to distinguish between ICH and drug-induced liver injury (DILI). Therefore, the detection of related liver function indices with PD-1 inhibitor therapy plays an important role in predicting survival. In both the training and the validation cohorts, the current nomogram shows good predictive accuracy of PFS, with a high C, NRI, and IDI indexes. This is expected to make the greatest prognostic contribution to the clinics using nomograms for the determination of risk factors that are related to liver function indicators.

There are some limitations to our study that need to be addressed. First, the analysis method of this study increases the possibility of bias. Further, some indicators, such as EBV-DNA, are good prognostic indicators of NPC. However, this was a retrospective analysis, and some patients were not routinely tested for EBV-DNA. Accordingly, we cannot know their prognosis following immunotherapy. Second, the sample size is insufficient and regional. If the sample size is large enough, the bias that is caused by the retrospective data collection may be reduced. Furthermore, although internal validation and calibration were performed to guarantee the nomogram’s universality, further validation of the nomogram using outside data and/or anticipated data sets is necessary to verify our results.

In summary, before administering PD-1 inhibitor therapy, we constructed and verified a nomogram for predicting PFS in NPC patients based on indicators related to liver function, and found that it improved the accuracy of prediction. Our nomogram can assist doctors in predicting the prognosis and survival of patients with NPC undergoing PD-1 inhibitor therapy. However, further validation in large-scale multi-center trials and prospective research are needed in the future to validate our findings.

## Figures and Tables

**Table 1:** Main clinical characteristics in 162 patients with NPC.

Characteristics	Total Cohort *N* (%)	Training Cohort *N* (%)	Validation Cohort *N* (%)
Gender			
Male	115 (71.0)	79 (73.1)	36 (66.7)
Female	47 (29.0)	29 (26.9)	18 (33.3)
Age (years)			
≤41	60 (37.0)	41 (38.0)	19 (35.2)
>41	102 (63.0)	67 (62.0)	35 (64.8)
BMI			
≤22.3	84 (51.9)	56 (51.9)	28 (51.9)
>22.3	78 (48.1)	52 (48.1)	26 (48.1)
Smoking history			
Non-smoker	121 (74.7)	80 (74.1)	41 (75.9)
Current or former smoker	41 (25.3)	28 (25.9)	13 (24.1)
Drinking history			
Non-drinker	143 (88.3)	98 (90.7)	45 (83.3)
Current or former drinker	19 (11.7)	10 (9.3)	9 (16.7)
Histological differentiation			
Undifferentiated	153 (94.4)	100 (92.6)	53 (98.1)
Poorly differentiated	9 (5.6)	8 (7.4)	1 (1.9)
Clinical stages			
II	11 (6.8)	9 (8.3)	2 (3.7)
III	46 (28.4)	36 (33.3)	10 (18.5)
IV	105 (64.8)	63 (58.4)	42 (77.8)
Tumor stage			
T0–T2	38 (23.5)	27 (25.0)	11 (20.4)
T3–T4	124 (76.5)	81 (75.0)	43 (79.6)
Node stage			
N0–N1	70 (43.2)	47 (43.5)	23 (42.6)
N2–N3	92 (56.8)	61 (56.5)	31 (57.4)
Metastasis stage			
M0	77 (47.5)	54 (50.0)	23 (42.6)
M1	85 (52.5)	54 (50.0)	31 (57.4)
Distant metastasis (One or multiple)			
Distant lymph nodes	63 (38.9)	46 (42.6)	17 (31.5)
Liver	26 (16.1)	17 (15.7)	9 (16.7)
Lung	36 (22.2)	21 (19.4)	15 (2.8)
Bone	45 (27.8)	28 (25.9)	17 (31.5)
Previous chemotherapy			
Radiotherapy	14 (8.6)	10 (9.2)	4 (7.5)
Chemotherapy	73 (45.1)	49 (45.4)	24 (44.4)
Chemoradiotherpy	75 (46.3)	49 (45.4)	26 (48.1)
Recurrence			
Yes	74 (45.7)	40 (37.0)	34 (62.9)
No	88 (54.3)	68 (63.0)	20 (37.1)
Outcomes			
CR	1 (0.6)	1 (0.9)	0
PR	52 (32.1)	45 (41.7)	7 (13.0)
SD	35 (21.6)	9 (8.3)	26 (48.1)
PD	74 (45.7)	53 (49.1)	21 (38.9)
PD-1 Blockade			
Camrelizumab	18 (11.1)	10 (9.3)	8 (14.8)
Toripalimab	119 (73.5)	81 (75.0)	38 (70.4)
Pembrolizumb	3 (1.9)	3 (2.8)	0
Sintilimab	22 (13.5)	14 (12.9)	8 (14.8)

**Table 2:** C-index for prediction of PFS in the training cohort and the validation cohort.

Factor	Training Cohort		Validation Cohort	
	C-Index (95%CI)	*p*	C-Index (95%CI)	*p*
Nomogram	0.732 (0.540–0.924)		0.847 (0.545–1.049)	
M	0.585 (0.397–0.773)		0.693 (0.455–0.930)	
ALT	0.625 (0.451–0.799)		0.632 (0.377–0.887)	
AST/ALT	0.641 (0.463–0.819)		0.656 (0.428–0.883)	
LDH	0.649 (0.473–0.825)		0.677 (0.436–0.918)	
TNM stage	0.617 (0.411–0.823)		0.727 (0.462–0.992)	
Nomogram vs. M		0.046		0.002
Nomogram vs. ALT		0.005		0.043
Nomogram vs. AST/ALT		0.002		0.007
Nomogram vs. LDH		<0.001		0.004
Nomogram vs. TNM stage		0.026		<0.001

**Table 3:** Predictive improvement of the nomogram in the training cohort and the validation cohort.

Factor	Training Cohort			Validation Cohort		
	NRI%	IDI%	*p*	NRI%	IDI%	*p*
Nomogram vs. M	22.3	17.6	<0.001	33.5	14.1	<0.001
Nomogram vs. ALT	27.7	16.5	<0.001	54.1	17.7	0.01
Nomogram vs. AST/ALT	32.2	10.3	<0.001	25.0	18.0	<0.001
Nomogram vs.LDH	25.7	13.5	<0.001	41.7	13.6	0.03
Nomogram vs. TNM stage	27.8	13.2	0.01	21.7	11.6	0.026

## Data Availability

On reasonable request, the corresponding authors will provide all the datasets used in this study.
